# One Virus, Four Continents, Eight Countries: An Interdisciplinary and International Study on the Psychosocial Impacts of the COVID-19 Pandemic among Adults

**DOI:** 10.3390/ijerph17228390

**Published:** 2020-11-13

**Authors:** Mélissa Généreux, Philip J. Schluter, Kevin KC Hung, Chi Shing Wong, Catherine Pui Yin Mok, Tracey O’Sullivan, Marc D. David, Marie-Eve Carignan, Gabriel Blouin-Genest, Olivier Champagne-Poirier, Éric Champagne, Nathalie Burlone, Zeeshan Qadar, Teodoro Herbosa, Gleisse Ribeiro-Alves, Ronald Law, Virginia Murray, Emily Ying Yang Chan, Nathalie Pignard-Cheynel, Sébastien Salerno, Grégoire Lits, Leen d’Haenens, David De Coninck, Koenraad Matthys, Mathieu Roy

**Affiliations:** 1 Department of Community Health Sciences, Faculty of Medicine & Health Sciences, Université de Sherbrooke, Sherbrooke, QC J1H 5N4, Canada; 2 School of Health Sciences, University of Canterbury-Te Whare Wananga o Waitaha, Christchurch 8140, New Zealand; philip.schluter@canterbury.ac.nz; 3 Collaborating Centre for Oxford University and CUHK for Disaster and Medical Humanitarian Response, JC School of Public Health and Primary Care, Chinese University of Hong Kong, Ngan Shing Street 30-32, Hong Kong SAR, China; kevin.hung@cuhk.edu.hk (K.K.H.); cswong@cuhk.edu.hk (C.S.W.); loncpym1@student.london.ac.uk (C.P.Y.M.); emily.chan@cuhk.edu.hk (E.Y.Y.C.); 4 Interdisciplinary School of Health Sciences, Faculty of Health Sciences, University of Ottawa, Ottawa, ON K1N 7K4, Canada; tosulliv@uottawa.ca; 5 Département de communication, Faculté des Lettres et Sciences Humaines, Université de Sherbrooke, Sherbrooke, QC J1K 2R1, Canada; marc.d.david@usherbrooke.ca (M.D.D.); marie-eve.carignan@usherbrooke.ca (M.-E.C.); olivier.champagne-poirier@usherbrooke.ca (O.C.-P.); 6 School of Applied Politics, Faculté des Lettres et Sciences Humaines, Université de Sherbrooke, Sherbrooke, QC J1K 2R1, Canada; gabriel.blouin-genest@usherbrooke.ca; 7 School of Political Studies, Faculty of Social Sciences, University of Ottawa, Ottawa, Ontario K1N 6N5, Canada; echampagne@uottawa.ca (É.C.); nburlone@uottawa.ca (N.B.); 8 National Collaborating Centre for Infectious Diseases, Rady Faculty of Health Sciences, University of Manitoba, Winnipeg, MB R3E 0T5, Canada; sheikh.qadar@umanitoba.ca; 9 Department of Emergency Medicine, College of Medicine, University of Philippines, Manille Grand Manille 1000, Philippines; ted.herbosa@gmail.com; 10 Centro Universitário de Brasília, Brasília 70850-090, Brazil; gleisse@yahoo.com; 11 Department of Health, Manila, Manille 2932, Philippines; ronlawmd@gmail.com; 12 Public Health England, London SE1 8UG, UK; virginia.murray@phe.gov.uk; 13 Académie du journalisme et des médias, Université de Neuchâtel, 2000 Neuchâtel, Switzerland; nathalie.pignard-cheynel@unine.ch; 14 Université de Genève, Boulevard du Pont-d’Arve 40, 1205 Genève, Switzerland; Sebastien.Salerno@unige.ch; 15 Institut Langage et Communication, Université catholique de Louvain, 1348 Louvain-la-Neuve, Belgium; gregoire.lits@uclouvain.be; 16 Institute for Media Studies, KU Leuven, 3000 Leuven, Belgium; leen.dhaenens@kuleuven.be; 17 Centre for Sociological Research, KU Leuven, 3000 Leuven, Belgium; david.deconinck@kuleuven.be (D.D.C.); koen.matthijs@kuleuven.be (K.M.); 18 Department of Family Medicine & Emergency Medicine, Faculty of Medicine & Health Sciences, Université de Sherbrooke, Sherbrooke, QC J1H 5N4, Canada; Mathieu.roy7@usherbrooke.ca

**Keywords:** pandemic, psychosocial impacts, sense of coherence

## Abstract

The novel coronavirus disease 2019 (COVID-19) pandemic brought about several features that increased the sense of fear and confusion, such as quarantine and financial losses among other stressors, which may have led to adverse psychosocial outcomes. The influence of such stressors took place within a broader sociocultural context that needs to be considered. The objective was to examine how the psychological response to the pandemic varied across countries and identify which risk/protective factors contributed to this response. An online survey was conducted from 29 May 2020–12 June 2020, among a multinational sample of 8806 adults from eight countries/regions (Canada, United States, England, Switzerland, Belgium, Hong Kong, Philippines, New Zealand). Probable generalized anxiety disorder (GAD) and major depression episode (MDE) were assessed. The independent role of a wide range of potential factors was examined using multilevel logistic regression. Probable GAD and MDE were indicated by 21.0% and 25.5% of the respondents, respectively, with an important variation according to countries/regions (GAD: 12.2–31.0%; MDE: 16.7–32.9%). When considered together, 30.2% of the participants indicated probable GAD or MDE. Several factors were positively associated with a probable GAD or MDE, including (in descending order of importance) weak sense of coherence (SOC), lower age, false beliefs, isolation, threat perceived for oneself/family, mistrust in authorities, stigma, threat perceived for country/world, financial losses, being a female, and having a high level of information about COVID-19. Having a weak SOC yielded the highest adjusted odds ratio for probable GAD or MDE (3.21; 95% confidence interval (CI): 2.73–3.77). This pandemic is having an impact on psychological health. In some places and under certain circumstances, however, people seem to be better protected psychologically. This is a unique opportunity to evaluate the psychosocial impacts across various sociocultural backgrounds, providing important lessons that could inform all phases of disaster risk management.

## 1. Introduction

The novel coronavirus disease 2019 (COVID-19) outbreak was declared by the World Health Organization (WHO) a global pandemic on 11 March 2020. Since its first identification among humans, more than 962,000 deaths from over 31.0 million cases across 215 countries have been reported as of 20 September 2020 [1]. National and community-level responses have varied markedly, as have the epidemiological sequelae. The profound effects of the COVID-19 pandemic on all aspects of society have led to a call for action for international collaborative research on mental health, including psychosocial evaluation, together with an understanding of the repeated media consumption and health messaging impact around the virus and pandemic context [2].

Psychosocial impacts have been globally observed during the pandemic. They could not only affected attention, understanding, and decision-making capacity, which could hinder the response against COVID-19 pandemic, but also produce a lasting effect on the wellbeing of individuals and communities. Recent studies found a consistent negative impact of COVID-19 pandemic on stress, anxiety, and depression in various countries [3]. A review of four Chinese studies found that 16% to 28% of respondents showed symptoms of anxiety and depression [4]. In Germany, a study involving over 6000 people showed that over 50% of the respondents expressed that they were suffering from anxiety and distress related to the COVID-19 pandemic [5]. In Italy and Spain, findings were also reported on similarly associated levels of mental stress [6,7]. Further findings from a survey conducted in Bangladesh showed that over 85% of the participants reported COVID-19-related stress, which resulted in disrupted sleep, short temper, and chaos in families [8]. Finally, in the United States of America (USA), an online study reported levels of depression and anxiety as high as 43.3% and 45.4% one month after the country declared a state of emergency [9].

As the pandemic continues to evolve, the interplay among communication strategies, media discourse, and psychosocial impacts is becoming evident. In the face of this unique global crisis co-occurring in a context where there is an unprecedented volume of media dissemination and consumption, little is known about the adverse effects that this combination may have on population mental health. A vast array of sources (multilevel health organizations, experts, policymakers, citizens) and channels (traditional, digital, interpersonal) are currently used to communicate information on COVID-19. Such rapid—and sometimes unqualified—information dissemination has been pointed out as a key feature that may exacerbate the negative psychosocial impacts of the pandemic [2]. Other features shared by the COVID-19 pandemic that may lead to adverse mental health outcomes are the sense of fear, worry, and uncertainty, coupled with additional stressors such as confusion, misinformation, mistrust, stigma, discrimination, disruptions, isolation, grief, and losses. The sense of coherence (SOC), a core concept of the salutogenic model [10], was proposed to explain why some people become sick under stress while others stay healthy. This construct expresses the degree to which a person is able to understand and integrate, to handle, and to make sense of everyday life stressors [10,11]. It may also be an important resource in mitigating the effects of the pandemic and coping with the unpredictable health threat imposed on individuals and societies [12].

These findings further support the need to recognize and understand the psychosocial impacts, their interplay with information sharing and use, and other challenges posed by the pandemic, in order to better manage public mental health around the world. While numerous studies in the current literature have confirmed that large-scale outbreaks adversely affect mental health [13] and that, in some places and under certain circumstances, people are better protected psychologically, there is limited understanding of the differences between sociocultural contexts in psychological response to pandemics and other disasters. Despite investing considerable effort and resources into identifying, tracking, and controlling the infection, as well as developing effective vaccines and treatments, the recognition of people whose mental state has worsened due to the pandemic has been comparatively partially neglected [14]. It is important to consider not only the physical and economic damages resulting from this pandemic, but also the state of mental health across the globe, its associated risk and protective factors, and the broader context in which they are embedded. In this regard, few studies attempted to understand the SOC’s ability to predict mental health outcome during the pandemic [12,15]. Most importantly, in this unique era, the full spectrum of traditional and COVID-19-specific stressors (e.g., confusion, misinformation) has rarely, if ever, been simultaneously explored, and even less so under various sociocultural circumstances.

In light of these findings, a number of scientific questions still need to be addressed, including how the population is reacting to the COVID-19 pandemic and how various stressors, including the media discourse (news media and social media), influence people’s reactions to the pandemic. The present study, therefore, aimed to examine the psychological response of populations from different countries and continents to the global COVID-19 crisis, in terms of anxiety and depression, and to identify individual factors positively or negatively contributing to this response.

## 2. Materials and Methods 

### 2.1. Design

This study took place within a broader research project funded by the Canadian Institutes of Health Research. It was reviewed and approved by the Research Ethics Board of the CIUSSS de l’Estrie—CHUS (HEC ref: 2020-3674). The overarching goal of this interdisciplinary and international research project was to better understand how the risk information is delivered and communicated by authorities and media, and how it is received, understood, and used by the public. Using a mixed-method approach, it was composed of three axes, including a repeated cross-sectional online survey, a discourse analysis of mainstream media and social media, and a network analysis to assess how official information flows and circulates across levels of governance. The current study fell within the first axis. The first data collection (i.e., the pilot phase) was conducted between 8 and 11 April 2020 (*n* = 600 Canadian adults), while Phase 1 of the international survey was conducted from 29 May 2020–12 June 2020, among a much larger sample of adults living in eight different countries or regions, from four continents. The eight countries/regions selected, namely, Canada, the United States of America (USA), England, Switzerland, Belgium, Hong Kong, Philippines, and New Zealand (NZ), represent a vast array of COVID-19 epidemiological situations and sociocultural backgrounds.

### 2.2. Selection of Participants

Recruitment and data collection were carried out by only two polling firms, with the collaboration of international partners, to ensure the standardization of the whole process. Any adults (≥18 years) living in each of the eight countries/regions listed above and able to answer an online questionnaire were eligible to participate in the online survey. Participants were randomly recruited from online panels. Several sources were used for the recruitment of panel members, including (a) random recruitment using traditional and mobile telephone methodologies, i.e., recruitment through the firm’s call center, and (b) recruitment by invitation, through social media (Facebook and Instagram), through offline recruitment, and through partner programs and campaigns such as the friend recommendation program. Significant efforts were made to maximize the representativeness of the sample by using software generating representative samples of the population and by including hard-to-reach groups through targeted recruitment. The final sample was composed of approximately 1000 adults per country/region, with the exception of Canada, which was oversampled to 1500 participants, in order to compare Quebec (the only French speaking province) to the rest of the country.

### 2.3. Data Collection

The elaboration of the data collection instrument (i.e., the online questionnaire) was based on the knowledge–attitude–practice (KAP) model [16] and, therefore, explored a wide range of aspects, going from risk perceptions and beliefs to positive/negative attitudes and adaptive/maladaptive behaviors. Sociodemographic characteristics were also assessed. The questionnaire contained closed-ended questions only and lasted an average of 18 min per participant. It was pretested and validated in five different languages (i.e., English, French, German, Italian, and Chinese).

### 2.4. Psychological Outcomes

Two psychological outcomes were assessed, including probable generalized anxiety disorder (GAD) and major depression episode (MDE), measured with the GAD-7 and the Patient Health Questionnaire-9 (PHQ-9) scales, respectively. These two scales are based on the diagnostic criteria for GAD and MDE described in DSM-IV. These seven- and nine-item questionnaires, respectively, were primarily designed for use by health professionals but they are also regularly used in population-based studies. The GAD-7 has a composite score ranging from 0–21, while the PHQ-9 score may range from 0–27. For both scales, combined sensitivity and specificity were shown to be maximized at a cutoff score of 10 or above, which is the standard cutoff used to identify moderate to severe symptoms of GAD or MDE [17,18]. A score of 10 or greater indicates a probable GAD or MDE that needs to be further evaluated by a clinician.

### 2.5. Potential Stressor Variables

Several factors previously identified in the literature as positively or negatively influencing the psychological response to the pandemic were examined (Table 1).

### 2.6. Sociodemographic Variables

Four sociodemographic variables were used, namely, gender (female, male), age (18–24, 25–34, 35–44, 45–54, 55–64, and ≥65 years), household composition (living alone, living with others including children, living with others but without children), and being an essential worker (e.g., healthcare and social services, law enforcement, emergency services, provider of essential goods). Although the education level was assessed through the questionnaire, it was not included in the international analyses as education systems differ widely among participating countries/regions.

### 2.7. Data Analysis

Reporting of analyses was informed by the STROBE guidelines (www.strobe-statement.org), and sampling weights (based on age, sex and region distribution) were used throughout. Initially, participant characteristics were described, partitioned by the participating countries. Treating countries as fixed effects, binomial regression models (with identity link function) were used to estimate rates of probable GAD, MDE, and GAD or MDE for each country, together with their associated 95% confidence intervals (CIs). Next, complete case multilevel mixed-effects logistic regression models were employed, treating countries as random intercept effects and participants nested within countries, to investigate the association between probable GAD or MDE indication and sociodemographic and potential stressor variables. The Bayesian information criterion (BIC) was used to establish whether this model characterization was better than a model treating countries as fixed effects. The BIC rewards goodness-of-fit and penalizes model complexity, with the preferred model yielding the lowest value of the criterion. Bivariable analyses were first conducted. All variables were utilized in pursuant multivariable models without selection. Two multivariable models were considered, as the Hong Kong survey included a subset of variables: one including Hong Kong participants and the second excluding them. The collective ability of the considered variables to predict probable GAD or MDE indication within these multivariable models was determined by a 10-fold cross-validated area under (AUC) the receiver operating characteristic (ROC) curve. An ROC curve provides a standardized way of evaluating the ability of a continuous marker to predict a binary outcome and plots the true positive rate (sensitivity) against a function of the false positive rate (1−specificity) at various levels of the marker [19]. An AUC of 0.5 suggests no discrimination, 0.7–0.8 is considered acceptable, 0.8–0.9 is considered excellent, and more than 0.9 is considered outstanding [20]. In k-fold cross-validation, the dataset is randomly partitioned into k approximately equally sized subsamples (or folds). At each iteration, one fold is retained as the validation data for testing the model and estimating the AUC, while the remaining k − 1 folds are used as training data for model estimation. This process is repeated k times, with each of the k folds used once as the validation data. K-fold cross-validation avoids the optimistic estimates of predictive performance known to exist when the full dataset is used for both model specification and prediction assessment. Finally, sensitivity analyses were conducted, using multiple imputation (MI) with chained equations (using M = 50 replications) for all variables within the multivariable models. Differences in estimated effect sizes between imputed and complete case analyses were reported. All analyses were conducted using Stata SE version 16.0 (StataCorp, College Station, TX, USA), and two-tailed α = 0.05 defined significance.

## 3. Results

### 3.1. Participants and Their Characteristics

The final sample consisted of 8806 adults (Canada: 1501; USA: 1065; England: 1041: Belgium: 1015; Switzerland: 1002; Hong Kong: 1140; Philippines: 1041; NZ: 1001). Overall, 51.9% were female, 46.1% were aged between 18–44 years, 33.4% lived in households with children, and 24.5% classified themselves as being essential workers. The weighted numbers for participants’ demographic characteristics appear in Table 2.

Significant differences were observed among countries/regions in the distributions of age, household composition, and essential worker (all *p* < 0.001), but not gender (*p* = 0.68). When comparing these values to the national statistics of each country, it can be stated that the samples are representative of the current sociodemographic situation of each region.

### 3.2. Psychological Outcomes

**Figure 1:**
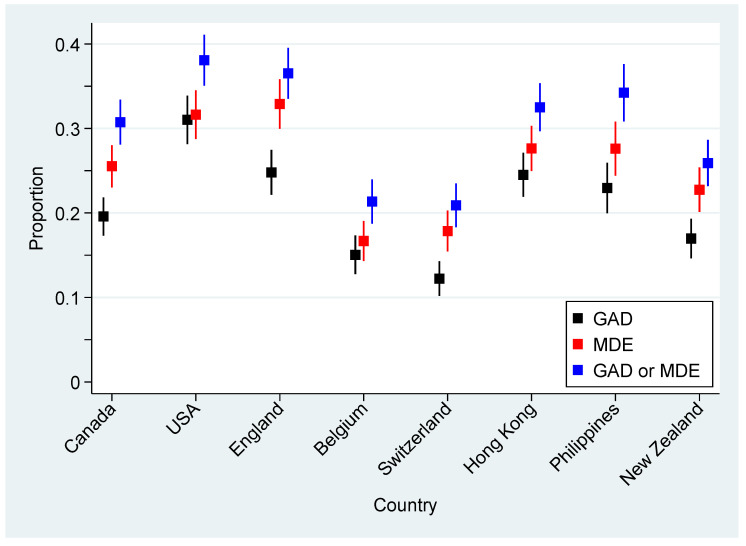
Proportion of participants indicated for probable generalized anxiety disorder (GAD), major depression episode (MDE), or either GAD/MDE, together with associated 95% confidence intervals (CIs), for the eight participating countries/regions.

Overall, probable GAD was indicated by 21.0% of participants while probable MDE was indicated by 25.5%. Significant variability was observed among countries/regions, with probable GAD ranging from 12.2% in Switzerland to 31.0% in the USA and probable MDE ranging from 16.7% in Belgium to 32.9% in England. Figure 1 presents these rates, together with 95% CIs, for the eight countries/regions. After adjusting for sex and age, these observed differences in indications among countries were still significant (both *p* < 0.001).

When considered together, 16.2% of participants qualified for both probable GAD and MDE, whereas 69.8% were not indicated for either. Among the discordant indications, 9.2% of participants were indicated for probable MDE but not GAD, while 4.8% were indicated for probable GAD but not MDE, an asymmetry that was significant (*p* < 0.001). Figure 1 also includes the rates for when either GAD or MDE was indicated, with a range of 20.9% in Switzerland to 38.1% in the USA.

### 3.3. Complete Case Multilevel Mixed-Effects Models

The intercept-only multilevel model of probable GAD or MDE was superior to the model treating countries as fixed effects (BIC: 10,687.21 vs. 10,709.39, respectively) and, thus, adopted hereafter. Table 3 includes the distribution of probable GAD or MDE for sociodemographic and potential stressor variables, together with bivariable and multivariable estimates derived from multilevel mixed-effects logistic models. Within these models, individuals were nested within countries/regions (treated as random intercepts). The estimated intraclass correlation (ICC) among participants within the same country was 0.026 (95% CI: 0.014, 0.049).

In bivariable analyses, all considered sociodemographic and potential stressor variables were significantly related to probable GAD or MDE indication (all *p* < 0.001, except gender *p* = 0.002) apart from the household composition (*p* = 0.06) and level of information about COVID-19 (*p* = 0.61), as presented in Table 3. In the multivariable model that included Hong Kong, all considered stressors were significant except for friend/family/coworkers as a regular source of information (*p* = 0.08). In this model, not having a strong SOC yielded the highest estimated adjusted odds ratio (aOR) for probable GAD or MDE indication (3.21; 95% CI: 2.73, 3.77) from the variables considered. In the second multivariable model, excluding Hong Kong, all considered stressors were significant apart from social networks used as a regular source of information (*p* = 0.27), and friend/family/coworkers as a regular source of information (*p* = 0.77). Again, the estimated effect size was highest for a strong SOC (3.13; 95% CI: 2.73, 3.59), as presented in Table 3.

**Figure 2:**
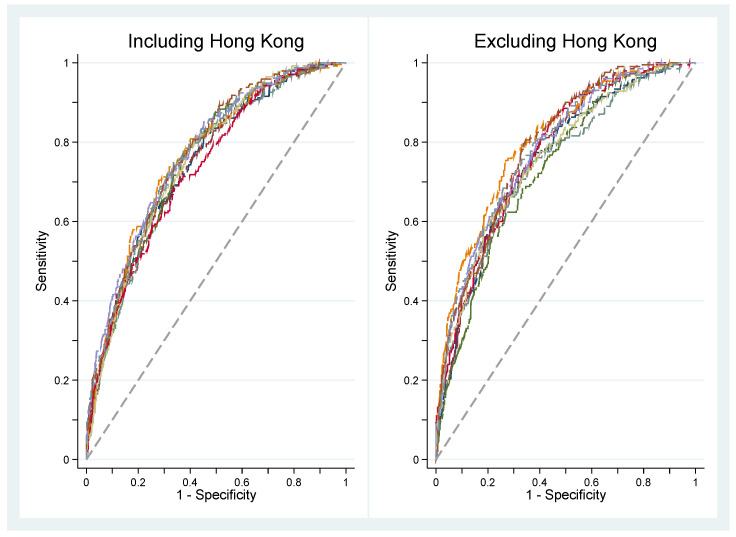
Tenfold cross-validation receiver operating characteristic curves for probable GAD or MDE derived from the adjusted complete case models characterized in Table 3.

Using 10-fold ROC curves derived from the multivariable models, the averaged cross-validated AUC was 0.756 (95% CI 0.743, 0.769) for the adjusted complete case multilevel logistic models which included Hong Kong and 0.770 (95% CI: 0.758, 0.782) when it was excluded. These represent acceptable predictive accuracy. Figure 2 depicts the 10-fold ROC curves derived from these models.

### 3.4. Sensitivity Analysis

After undertaking chained equations MI for missing data, the median difference in estimates between the complete case and MI models in the analyses including Hong Kong was 0.00, ranging from −0.14 for the age variable (25–34 years: complete case aOR = 2.61, MI aOR = 2.75) to 0.09 for the financial loss variable (unsure/unknown: complete case aOR = 1.50, MI aOR = 1.41). In the analyses excluding Hong Kong, the median difference was 0.01, ranging from −0.17 for the age variable (25–34 years: complete case aOR = 2.74, MI aOR = 2.91) to 0.21 for the false beliefs score variable (Q4: complete case aOR = 2.55, MI aOR = 2.34). In all cases, the overlap on 95% CIs was moderate to high between complete case and MI-derived estimates.

## 4. Discussion

Findings from this large international study strongly suggest that the current pandemic is having significant impacts on psychological health across the globe. Probable GAD or MDE was indicated by almost one-third of respondents (30.2%), with significant variation among the eight participating countries/regions (range 20.9–38.1%) and a modest ICC between participants within countries. Interestingly, the epidemiological situation in each jurisdiction did not seem to be associated with these psychological outcomes. Indeed, two of the four countries with the highest cumulative incidence of COVID-19 at the end of May 2020 (i.e., Belgium and Switzerland) appeared to be the least psychologically affected by the crisis.

Levels of anxiety and depression in the eight countries/regions were disturbingly elevated, as observed in previous studies [4,5,6,7,8,9]. In a pilot survey conducted among 600 adults in Canada from 8–11 April 2020 (i.e., peak of the first wave of the pandemic in Canada), 25.4% of respondents presented symptoms compatible with generalized anxiety [21]. Two months later, the proportion of probable GAD was lowered to 19.6% in this country (according to data from Phase 1 of the international survey), but remained substantially higher than what was estimated in the pre-pandemic era (i.e., 2.5% [22]). The same applies to the current level of major depression in Canada, which may be four times higher than before the pandemic [23]. For a better comparison, the actual estimated prevalence of generalized anxiety and major depression among Canadian adults may be similar to, if not higher than, that observed in the community of Fort McMurray (Canada) six months after the devastating 2016 wildfires (19.8% and 14.8%, respectively [24,25]).

This study builds on the limitations of previous studies by including countries or regions at different stages of the pandemic and with very different political, social, and economic backgrounds, while also taking into account a large set of classic and “newer” stressors hypothesized to influence mental health in times of the current pandemic. Several sociodemographic, psychological, and sociocultural factors were found to be positively associated with a probable GAD or MDE, including (in descending order) weak SOC, lower age, false beliefs, isolation, threat perceived for oneself or family, mistrust in authorities, being the victim of stigma, threat perceived for country or world, financial losses, being a female, and high level of information about COVID-19. Of all the potential factors considered, the SOC yielded the highest association with probable GAD or MDE, with respondents who had a weak SOC being three times more likely to display moderate to severe symptoms of anxiety or depression than those with a stronger SOC.

Perhaps the most important finding emerging from this study is precisely the key role that the SOC plays in predicting common psychopathological symptoms in the face of adversity. People who have developed a strong SOC over their lifetime have the capacity to effectively deal with stressful circumstances. As a result, SOC appears to be a very important and apparently underestimated resource in minimizing the psychosocial impacts of the pandemic. This novel finding stresses the need for addressing individual recovery as part of a community recovery approach. An emerging literature seeks an asset-based approach to foster engagement, meaning-making, goal-setting, empowerment, and problem-solving, at both the individual and the community levels [26].

Some of the factors found in this study to be associated with decreased psychological health in times of the pandemic were also reported in previous research, notably, female gender, younger age, discrimination, isolation, and lower adaptive capacity [27,28,29]. Beyond factors classically associated with post-traumatic stress disorder, anxiety, or depression during and after disasters (including large-scale outbreaks), our study found that false beliefs, mistrust in authorities, and using social media or the close social circle (e.g., family, friends, coworkers) as regular sources of information about the COVID-19 pandemic may all have shaped negatively psychological responses to the current pandemic. The relationships between using informal sources of information (e.g., social media and close social circle) and psychological health were, however, strongly attenuated (and even disappeared according to models) when considering other stressor variables. Reporting a high level of information about the COVID-19 pandemic, in contrast, became a significant stressor in multivariable analyses. This latter observation needs to be explored more thoroughly in future studies. This may suggest that it is not the quality of the sources of information that matters most, but rather the quantity of sources used, which may lead in some cases to media overexposure, information overload, and ultimately stress, distress, and sadness.

This study has strengths that overcome important limitations observed in previous studies, including a standardized methodology and instrument using largely psychometrically robust tools, a concurrent administration in several countries, a short survey window best suited for capturing a rapidly evolving situation, and a large sample size. It also has some limitations that must be underscored. First, its cross-sectional nature precludes the inference of causality between stressors and psychological outcomes. Second, the use of an online questionnaire may have impaired the representativeness of the sample, with adults who cannot read and those who are less comfortable using a computer being potentially underrepresented. Third, our study is based on self-reported measures (including scales designed for use by health professionals) which may be subject to information bias. While anxiety and depression symptoms were assessed using scales possessing good psychometric properties, many measures of potential stressors have not been validated previously. Furthermore, the education level was not included in the international analyses due to comparability issues. Future studies could compare this factor using the International Standard Classification of Education as this variable can affect comprehension and access to information. Finally, some stressors or potential confounders may not have been measured. For example, several country-specific characteristics (e.g., the COVID-19 epidemiological situation, inequality indicators such as the Gini coefficient) were not included in the regression models. Future work should consider meso- and macrolevel variables that can be incorporated from external datasets (e.g., the World Bank). 

## 5. Practical Implications of the Results 

While charting the epidemiological psychosocial sequelae of the COVID-19 pandemic across countries, large and substantial variations in mental disorder symptoms were uncovered. Despite this, after controlling for country and sociodemographic differences, common important features influencing the psychological response to the pandemic were identified. Being aware of these features is essential in developing health-promoting interventions and social measures. According to our findings, such interventions should attempt to strengthen the SOC, promote accurate communication channels, and develop communication strategies contributing to a climate of trust and reducing confusion and misinformation. These elements should be included in authorities’ responses to COVID-19 (and future health emergencies) at the local, national, and international levels, strengthening SOC at multiple levels. 

## 6. Conclusions

There is no doubt that the COVID-19 pandemic is taking a toll on the mental health of billions of people from all four corners of the globe. Future research should further explore how the SOC shapes the psychological reaction to pandemics and which interventions may take advantage of this relation. The current pandemic represents a unique opportunity to evaluate the associated psychological impacts in various sociocultural groups and contexts, providing important lessons that could be applied in response to future disasters.

## Figures and Tables

**Table 1:** Risk and protective factors assessed in the international online survey (Phase 1, 29 May 2020–12 June 2020).

Variables Names	Descriptions and Response Options
Having experienced self-isolation/quarantine	Having experienced self-isolation/quarantine, mandatory or voluntary (yes because of symptoms or diagnosis of the novel coronavirus disease 2019 (COVID-19), yes for other reasons, no).
Having experienced financial losses	Having experienced financial losses of any kind due to the COVID-19 (yes, no).
Threat perceived for oneself and/or family	Level of threat posed by the COVID-19 perceived for oneself and/or the family (very low/low/moderate, high/very high).
Threat perceived for country and/or world	Level of threat posed by the COVID-19 perceived for the country and/or the world (very low/low/moderate, high/very high).
Being a victim of stigma	Being a victim of stigma or discrimination due to the COVID-19 (yes, no).
Level of information about COVID-19	Level of information about the coronavirus, with a scale ranging from 1 to 10 (high (9, 10), lower level (0–8)).
Level of trust in authorities	Level of trust in authorities (scientists, doctors, and health experts; national health organizations; global health organizations; government), each with a scale ranging from 1 to 10. The sum of these four distinct scores (total score ranging from 4–40) was then divided into quartiles.
False beliefs score	False beliefs score based on 12 statements scientifically unfounded (e.g., “I believe the coronavirus was made intentionally in a laboratory”, or “I believe the coronavirus is not transmitted in warm countries”). Participants had to agree on a scale ranging from 1–10 on each of these statements. The sum of these 12 scores (total score ranging from 12–120) was then divided into quartiles.
Sources regularly used	Sources regularly used to get informed about the COVID-19 including the World Health Organization (WHO), government, public health authorities, health professionals, news media (television, radio, newspapers), friend, family and coworkers, social networks, and the Internet. Respondents had to report the frequency of use, which was subsequently dichotomized as “a lot/somewhat” vs. “not much/not at all”, for each source of information.
Sense of coherence (SOC)	Sense of coherence (SOC) measured with a three-item questionnaire (i.e., SOC-3) that was developed for the needs and constraints of large population studies and that has shown adequate psychometric properties [11]. Each question corresponded to one of the three components of the SOC. The total score, which ranged from 0–6, was dichotomized using a standard threshold (weak (0–4) or strong (5, 6) SOC).

**Table 2:** Weighted numbers for the demographic characteristics of participants. USA, United States of America; NZ, New Zealand.

Demographic Characteristics	Canada		USA		England		Belgium		Switzerland		Hong Kong		Philippines		NZ	
	*n*	(%)	*n*	(%)	*n*	(%)	*n*	(%)	*n*	(%)	*n*	(%)	*n*	(%)	*n*	(%)
Sex *																
Female	722.5 (48.4)		516.4 (48.5)		507.9 (48.8)		493.7 (48.6)		477.5 (47.7)		513.2 (45.1)		509.9 (49.2)		484.2 (48.6)	
Male	771.8 (51.6)		548.4 (51.5)		531.9 (51.2)		521.1 (51.4)		522.5 (52.3)		625.8 (54.9)		521.9 (50.6)		512.2 (51.4)	
**Age (years)**																
18–24	163.0 (10.9)		58.9 (5.5)		115.8 (11.1)		62.5 (6.2)		95.5 (9.5)		108.3 (9.5)		224.4 (21.6)		122.3 (12.2)	
25–34	246.6 (16.4)		225.5 (21.2)		181.2 (17.4)		207.8 (20.5)		144.1 (14.4)		196.2 (17.2)		260.3 (25.0)		184.3 (18.4)	
35–44	242.7 (16.2)		191.1 (17.9)		170.0 (16.3)		139.5 (13.7)		138.3 (13.8)		205.8 (18.1)		208.9 (20.1)		162.8 (16.3)	
45–54	269.1 (17.9)		203.6 (19.1)		185.9 (17.9)		210.3 (20.7)		176.8 (17.6)		218.2 (19.1)		161.5 (15.5)		175.3 (17.5)	
55–64	262.4 (17.5)		189.5 (17.8)		151.2 (14.5)		171.5 (16.9)		239.1 (23.9)		201.7 (17.7)		106.0 (10.2)		157.5 (15.7)	
≥65	317.2 (21.1)		196.4 (18.4)		236.9 (22.8)		223.5 (22.0)		209.3 (20.8)		209.8 (18.4)		79.8 (7.7)		198.9 (19.9)	
**Household composition**																
Alone	303.7 (20.2)		233.2 (21.9)		215.2 (20.7)		192.1 (18.9)		236.1 (23.6)		74.3 (6.5)		49.7 (4.8)		180.6 (18.0)	
Children	409.4 (27.3)		375.1 (35.2)		308.0 (29.6)		278.9 (27.5)		264.9 (26.4)		493.8 (43.3)		462.0 (44.4)		349.5 (34.9)	
Other	788.0 (52.5)		456.7 (42.9)		517.8 (49.7)		542.8 (53.5)		501.1 (50.0)		571.9 (50.2)		529.3 (50.8)		471.0 (47.0)	
**Essential worker**																
Yes	357.2 (24.1)		229.9 (21.8)		274.9 (26.9)		176.5 (18.8)		222.9 (22.8)		400.0 (35.7)		184.7 (18.8)		267.9 (27.1)	
No	1125.5 (75.9)		823.8 (78.2)		748.5 (73.1)		816.7 (82.2)		756.8 (77.2)		721.1 (64.3)		797.6 (81.2)		722.0 (72.9)	

* Eighteen participants declared a gender identity that was neither female nor male and a further seven participants preferred not to answer the question. These 25 participants had their sex set to missing.

**Table 3:** Distribution of probable GAD or MDE for sociodemographic and potential stressor variables, together with estimated crude and adjusted complete case multilevel logistic model odds ratios (ORs) and associated 95% confidence intervals (CIs).

		GAD or MDE		Crude		Adjusted ^1^		Adjusted ^2^	
	*N*	*n*	(%)	OR	(95% CI)	aOR	(95% CI)	aOR	(95% CI)
**Sex**									
Female	4555.6	1470.9	(32.3)	1.24	(1.09, 1.42)	1.34	(1.20, 1.50)	1.24	(1.07, 1.44)
Male	4225.4	1175.1	(27.8)	1	(reference)	1	(reference)	1	(reference)
**Age (years)**									
18–24	950.8	422.9	(44.5)	4.27	(2.74, 6.65)	3.38	(2.81, 4.07)	3.00	(2.16, 4.17)
25–34	1646.0	699.3	(42.5)	3.88	(2.59, 5.82)	2.74	(2.16, 3.46)	2.61	(1.91, 3.56)
35–44	1459.0	543.6	(37.3)	3.07	(2.06, 4.57)	2.37	(1.97, 2.84)	2.13	(1.60, 2.85)
45–54	1600.7	461.1	(28.8)	2.12	(1.60, 2.80)	1.96	(1.64, 2.35)	1.71	(1.33, 2.21)
55–64	1478.7	263.6	(17.8)	1.14	(0.75, 1.72)	1.35	(1.15, 1.59)	1.10	(0.75, 1.61)
≥65	1670.7	270.2	(16.2)	1	(reference)	1	(reference)	1	(reference)
**Household composition**									
Alone	1484.9	391.0	(26.3)	1	(reference)	1	(reference)	1	(reference)
Children	2941.5	968.5	(32.9)	1.32	(1.00, 1.74)	1.07	(0.89, 1.29)	1.17	(0.95, 1.4)
Other	4378.6	1300.0	(29.7)	1.17	(1.01, 1.35)	0.95	(0.88, 1.02)	0.97	(0.88, 1.07)
**Essential worker**									
No	6512.1	1803.0	(27.7)	1	(reference)	1	(reference)	1	(reference)
Yes	2113.9	793.2	(37.5)	1.56	(1.27, 1.91)	1.07	(0.93, 1.24)	1.21	(1.01, 1.43)
**Self-isolation/quarantine**									
No	3182.5	719.5	(22.6)	1	(reference)	1	(reference)	1	(reference)
Yes, case/symptom-free	4387.1	1317.0	(30.0)	1.46	(1.28, 1.67)	1.38	(1.21, 1.57)	1.31	(1.15, 1.49)
Yes, case or symptoms	949.4	491.2	(51.7)	3.60	(2.58, 5.04)	2.16	(1.62, 2.87)	2.27	(1.74, 2.97)
**Financial losses**									
No	3743.0	854.7	(22.8)	1	(reference)	1	(reference)	1	(reference)
Yes	4008.6	1441.8	(36.0)	1.83	(1.63, 2.06)	1.35	(1.12, 1.64)	1.36	(1.17, 1.58)
Unsure/unknown	1054.4	364.1	(34.5)	1.91	(1.64, 2.22)	1.46	(1.19, 1.79)	1.50	(1.25, 1.80)
**Threat perceived for oneself and/or family**									
High	2967.5	1278.6	(43.1)	2.43	(2.08, 2.84)	1.99	(1.83, 2.17)	2.12	(1.91, 2.36)
Otherwise	5569.5	1297.9	(23.3)	1	(reference)	1	(reference)	1	(reference)
**Threat perceived for country and/or world**									
High	6223.2	2050.7	(33.0)	1.65	(1.40, 1.94)	1.43	(1.26, 1.63)	1.31	(1.19, 1.45)
Otherwise	2284.8	524.5	(23.0)	1	(reference)	1	(reference)	1	(reference)
**Being a victim of stigma**									
No	6607.1	1700.0	(25.7)	1	(reference)	1	(reference)	1	(reference)
Yes	1254.7	615.1	(49.0)	2.71	(2.11, 3.49)	1.45	(1.21, 1.73)	1.57	(1.28, 1.92)
Decline to answer	944.1	345.5	(36.6)	1.67	(1.38, 2.03)	1.17	(0.95, 1.43)	1.30	(1.08, 1.56)
**Level of information about COVID-19**									
High (9–10)	2784.5	840.0	(30.2)	1	(reference)	1	(reference)	1	(reference)
Otherwise (1–8)	6021.5	1820.6	(30.2)	1.03	(0.93, 1.13)	0.85	(0.77, 0.95)	0.86	(0.78, 0.94)
**Trust in authorities score**									
Q1 (low)	2361.8	828.6	(35.1)	1.59	(1.06, 2.39)	1.66	(1.21, 2.27)	1.83	(1.34, 2.51)
Q2	2121.9	665.0	(31.3)	1.37	(1.16, 1.61)	1.36	(1.29, 1.44)	1.41	(1.33, 1.49)
Q2	2166.3	602.8	(27.8)	1.13	(0.92, 1.39)	1.21	(0.98, 1.50)	1.23	(0.99, 1.52)
Q4 (high)	2156.0	564.3	(26.2)	1	(reference)	1	(reference)	1	(reference)
False beliefs score *									
Q1 (low)	1987.6	382.0	(19.2)	1	(reference)	1	(reference)	-	-
Q2	1900.7	437.3	(23.0)	1.29	(1.12, 1.48)	1.17	(1.07, 1.27)	-	-
Q3	1931.5	572.0	(29.6)	1.82	(1.33, 2.50)	1.52	(1.14, 2.02)	-	-
Q4 (high)	1846.2	898.6	(48.7)	4.08	(2.69, 6.17)	2.55	(1.87, 3.46)	-	-
**Social networks used as a regular source of information**									
Often/always	2685.1	1054.5	(39.3)	1.79	(1.42, 2.24)	1.09	(0.94, 1.28)	1.16	(1.00, 1.35)
Sometimes/never	5780.9	1522.6	(26.3)	1	(reference)	1	(reference)	1	(reference)
**Friend/family/coworkers as a regular source of information**									
Often/always	3514.5	1232.7	(35.1)	1.42	(1.26, 1.60)	1.02	(0.88, 1.19)	1.11	(0.99, 1.25)
Sometimes/never	5113.5	1374.7	(26.9)	1	(reference)	1	(reference)	1	(reference)
**Sense of coherence**									
Strong (5–6)	2649.7	357.2	(13.5)	1	(reference)	1	(reference)	1	(reference)
Weak (0–4)	6156.3	2303.4	(37.4)	3.80	(3.16. 4.57)	3.13	(2.73, 3.59)	3.21	(2.73, 3.77)

* Data not collected in Hong Kong; ^1^ excluding Hong Kong (*n* = 6776); ^2^ including Hong Kong (*n* = 7819).
